# Jointless Bioinspired Soft Robotics by Harnessing Micro and Macroporosity

**DOI:** 10.1002/advs.202302080

**Published:** 2023-06-15

**Authors:** Seonggun Joe, Ouriel Bliah, Shlomo Magdassi, Lucia Beccai

**Affiliations:** ^1^ Soft BioRobotics Perception Istituto Italiano di Tecnologia (IIT) Genova 16163 Italy; ^2^ Casali Center for Applied Chemistry Institute of Chemistry Center for Nanotechnology and Nanoscience Hebrew University of Jerusalem Jerusalem 9190401 Israel; ^3^ Singapore‐HUJ Alliance for Research and Enterprise (SHARE) Smart Grippers for Soft Robotics (SGSR) Campus for Research Excellence and Technological Enterprise (CREATE) Singapore 138602 Singapore

**Keywords:** elephant trunks, porous materials, programmed motion, soft robotics, volumetric tessellation

## Abstract

Although natural continuum structures, such as the boneless elephant trunk, provide inspiration for new versatile grippers, highly deformable, jointless, and multidimensional actuation has still not been achieved. The challenging pivotal requisites are to avoid sudden changes in stiffness, combined with the capability of providing reliable large deformations in different directions. This research addresses these two challenges by harnessing porosity at two levels: material and design. Based on the extraordinary extensibility and compressibility of volumetrically tessellated structures with microporous elastic polymer walls, monolithic soft actuators are fabricated by 3D printing unique polymerizable emulsions. The resulting monolithic pneumatic actuators are printed in a single process and are capable of bidirectional movements with just one actuation source. The proposed approach is demonstrated by two proof‐of‐concepts: a three‐fingered gripper, and the first ever soft continuum actuator that encodes biaxial motion and bidirectional bending. The results open up new design paradigms for continuum soft robots with bioinspired behavior based on reliable and robust multidimensional motions.

## Graphical abstract

A novel design approach is introduced for continuum soft multidimensional actuation, based on both material microporosity, and on volumetric tessellation (macroporosity) design. Monolithic soft actuators result from a single process 3D printing of highly stretchable foams enveloped by a soft skin. A new generation of pneumatic elastic lattice actuators with programmed deformations and with smooth stiffness gradients is developed.

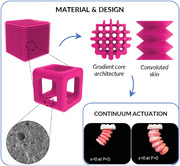

## 1 Introduction

Soft robotics is outperforming rigid body robotics through new approaches to implement known or new robotic tasks such as grasping, locomotion, climbing, and growing.^[^ ^1^ ^]^ Totally new bioinspired designs are possible because of the inherent compliance and deformability of the robot bodies,^[^ ^2^ ^]^ which much like living organisms, can reach the versatility needed to adapt to the real‐world.

The boneless elephant trunk, being a natural continuum universal manipulator, is a remarkable source of inspiration. Based on its amazing agility, this organ without joints is capable of delicate and precise, yet strong, grasping and manipulation.^[^ ^3^ ^]^ Recently it has been deciphered how the coordinated contractions of antagonist muscles in trunk enable different movements (e.g., contraction, elongation, bending, and stiffening) mainly due to the near‐incompressibility of the self‐supporting trunk tissues.^[^ ^3a^ ^]^ The intricate and unique composition of muscle fibers, fat, connective tissues, blood vessels, nerves, etc., presents a mechanical continuity that appears to disobey the well‐known kinematic principles (i.e., based on articulated skeletons), with an architecture that is naturally programmed for multidimensional motions.^[^ ^3a^ ^]^

The elephant trunk inspires innovative design principles, technologies, and materials, for developing new versatile manipulators with no distinction between arm and gripper. In this vision, a major unmet challenge in soft robotics is thus to develop actuated soft (i.e., boneless) structures that are highly deformable and reliable, with no sharp distinction in stiffness, in which different movements can be programmed as a result of the used materials and design of the core structure.

Multidimensional actuators have been developed by compromising several approaches,^[^ ^4^ ^]^ such as soft actuators built from different materials exhibiting independent movements (i.e., axial, bending, and torsion) via discretely distributed stiffness or Poisson's ratio.

However, the interfacing among the various functional and mechanical parts is not smooth, thus failing to achieve the desired kinematic trajectories, and repeatability and reliability are limited. Moreover, although fiber reinforcement has been used for soft pneumatic artificial muscles (PAMs) given that they improve actuation stiffness, localized deformation, and robustness,^[^ ^5^ ^]^ the fabrication is complicated since the fibers require winding (often manual) along the actuators.

Other methods involving cellular materials (e.g., foam‐like) offer good design flexibility, while allowing for desired deformations and enhanced stiffness.^[^ ^6^ ^]^ However, to the best of knowledge, so far only uniaxial movements, without programmed deformation, have been developed.

In contrast, metamaterial‐based approaches enable programming mechanical properties in monolithic structures.^[^ ^7^ ^]^ The overall density can thus be reduced, thereby obtaining compliant mechanisms. High resistance to both shear deformation and indentation can be implemented, which is essential for designing continuum structures,^[^ ^8^ ^]^ with high energy absorption promoting energy‐efficient designs.^[^ ^9^ ^]^

To date, mainly passive (non‐actuated) structures have been developed by 3D architected materials, for example, with encoded buckling by means of unit Voronoi tessellation,^[^ ^10^ ^]^ in an adaptable soft gripper,^[^ ^11^ ^]^ and in a fin ray structure for gentle grasping.^[^ ^12^ ^]^ Also, in a pioneering study a chemically fabricated porous material was demonstrated in a bending pneumatic actuator.^[^ ^13^ ^]^ However, due to the limited inflation ratio of the porous network, the internal structure fails when overinflated. A possible solution for this problem is through an architecture in the form of volumetric tessellations. They are currently manufactured using casting^[^ ^14^ ^]^ and lithography‐assisted pre‐patterning or etching,^[^ ^15^ ^]^ however, they result in limited 3D structural complexity and stretchability.

**Figure 1:**
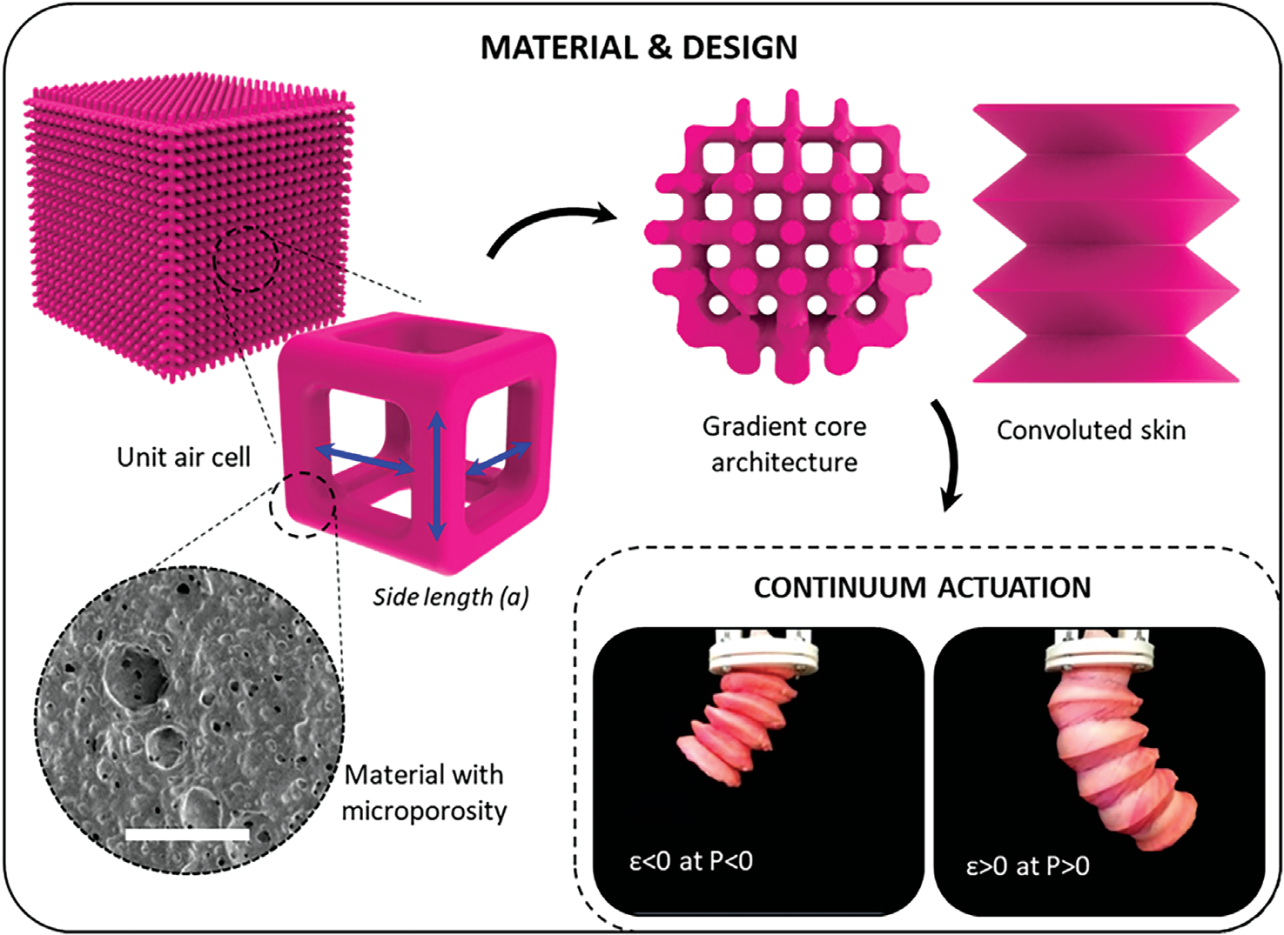
Novel design approach for continuum multidimensional actuation by harnessing porosity at both levels of material (microporosity) and design (macroporosity). The scale bar of the SEM image is 50 µm.

In this work we present a new design approach for soft multidimensional actuation, where porosity is created in the material by microporosity, and by design via volumetric tessellated structures (i.e., macroporosity), by 3D printing complex stretchable foams enveloped by a soft skin. We have thus created a new generation of pneumatic elastic lattice actuators (PELAs) with programmed deformations and with smooth stiffness gradients (**Figure** **1**).

The process is based on new printing compositions composed of a water‐in oil emulsion, while the continuous phase results in a highly stretchable polymer after printing, when the water droplets evaporate and form a porous structure with interconnected air voids (microporosity). The 3D printing produces objects with gradually dimensioned lattice structures, with micropores integrated into the walls.

Due to this structure, the kinematic movements of the printed actuators are determined by the total equivalent stiffness obtained from a combination of isotropy and anisotropy of the unit cells. The convoluted skin transforms the passive core structure in the actuator by ensuring high biaxial deformability, while avoiding structure instability. The mechanical behavior of the architected material leads to new design principles with globally or locally distributed stiffness, while the fabrication is performed by a single‐step 3D printing process using a commercial digital light processing (DLP) printer.^[^ ^16^ ^]^ The new printing compositions that were utilized are based on emulsion, which can be printed via a photolithography‐based printing process,^[^ ^17^ ^]^ while the continuous phase of the emulsion is made up of photopolymerizable stretchable polyurethane.^[^ ^18^ ^]^

In order to demonstrate the potential of this approach, two proof‐of‐concepts are presented: soft grippers with programmed structures, capable of compliant shape‐adaptation to objects having various shapes; and the first continuum actuator (without any joints) encoding axial and bending movements within the same structure.

## 2 Results

### 2.1 Mechanical Behavior

The mechanical behavior of 3D printed tensile specimens that are obtained from the new emulsion ink (that embed 2 µm micropores) was evaluated by tensile testing and by implicit constitutive modeling using nonlinear numerical interpolations. In general, this method can be applied for any soft material, as long as it is immiscible with water, and a suitable surfactant is found. For example, in a previous paper we reported on oil‐in‐water emulsion formulation for 3D printing that resulted in polymeric, though rigid, conductive 3D porous structures.^[^ ^19^ ^]^

**Figure 2:**
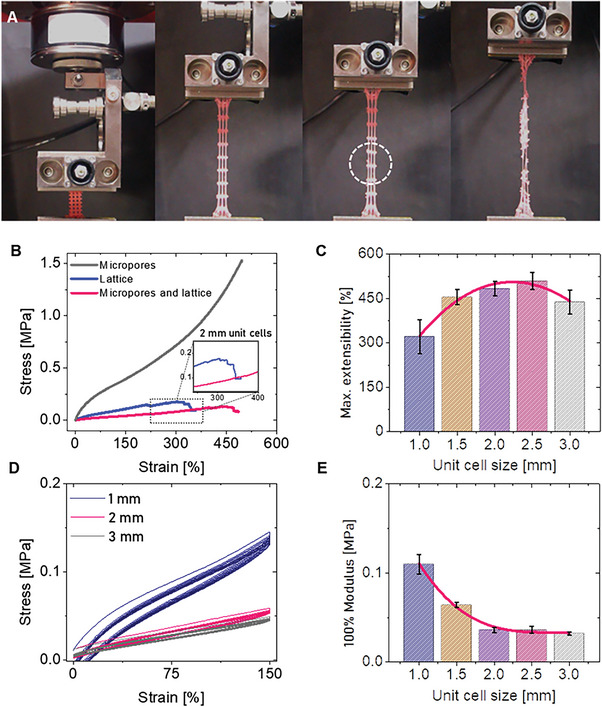
Tensile test results. A) Sequential photographs of the sample having, both, micropores (2 µm), and macropores (2 × 2 × 2 mm) at different extension phases. From left to right: initial state, reversible extensibility state (363% strain), first breaking point (white dot circle), and full break. B) Mechanical characteristics of samples consisting of printed emulsion ink (EI) with micropores (gray line), printed lattice structure (2 mm) without (blue line) and with (pink line) 2 µm micropores, respectively. C) Maximum extensibility versus unit cell size. D) Cyclic extension tests at 150% strain. E) 100% modulus versus unit cell size.

The results show a high deformability, up to 523.09% (std 27.17) with a tensile strength of 1.57 MPa (std 0.07) (**Figure** **2**B). The porosity can be controlled by a variety of parameters, which affect the mechanical properties of the bulk material. For example, emulsions prepared by high shear mixing produced pores with average size of 7 µm, compared to 2 µm for the emulsions prepared by homogenization, and density of 1.3 and 1.1 g cm^−3^, respectively. The mechanical properties are different in the two cases, with tensile strength and strain decreased of 49% and 20%, accordingly. 3D architected samples were designed and fabricated to investigate the influence of the lattice topology on both extensibility and compressibility (not achievable in bulk materials), which are fundamental for the design of multidimensional actuation. To explore possible design principles, a finite element modeling (FEM) analysis was used. The analytical models fit the experimental curve very well (see Table S1, Supporting Information, for the derived parameters).

#### 2.1.1 Extensibility

In Figure S1A–E, Supporting Information, the results of the tensile tests are shown for samples with different unit air cells ranging from 1 to 3 mm. Table S2, Supporting Information, presents a comparative analysis with respect to the bulk material. The tessellated structures have a limited maximum extensibility (down to 38.6%), and tensile and yield stresses (down to 93.4% and 96.2%, respectively). In addition, the maximum extensibility increases, as dimensions of the unit cells increase (Figure 2C).

There is a 41.4% variation in extensibility between samples with unit cell side of 1 and 1.5 mm, which is remarkable. In fact, material microporosity improves deformation and reduces stiffness. As shown in Figure 2B, in lattice samples (2 mm unit cells) without the presence of micropores in the unit cells’ walls, there is less deformability (up to 400.77%) and larger tensile strength (0.15 MPa) than in samples where microporosity is created in the emulsion ink. The latter extends up to 493% and presents a tensile strength of 0.13 MPa. On the other hand, 100% modulus and yield stress responses behave oppositely from the strain; the 100% modulus decreases from 0.11 to 0.03 MPa, as the air cell dimension increases from 1 to 3 mm. For structures with 3 mm air cells, the reversible extensibility (261.83%) shows a 40.3% reduction with respect to the maximum extensibility (438.5%). Similarly, the yield stress varies from 0.19 MPa (1 mm unit cell) to 0.066 MPa (3 mm unit cell).

From a dynamic point of view, during a cyclic extension test at 150%, a hysteresis ranging from 5% (1 mm) to 2% (3 mm) can be observed, as shown in Figure 2D long‐term cyclic tensile and compression tests (see Supporting Information text and Figure S3, Supporting Information) are performed to identify the safe operation range. For tensile stresses below 0.4 MPa (corresponding to 150% tensile strain) more than 1000 cycles can be ensured.

In summary, the results show that the stiffness can be tuned within a wide span, that is, up to 243%, at the 100% modulus (Figure 2E). Given the reversible extensibility, the structural deformation that takes place before the extension can be limited up to 370%. In addition, the extension can be limited up to 150%, so that the desired architecture can be frustration‐free while avoiding material failures due to repeated operations.

#### 2.1.2 Compressibility

**Figure 3:**
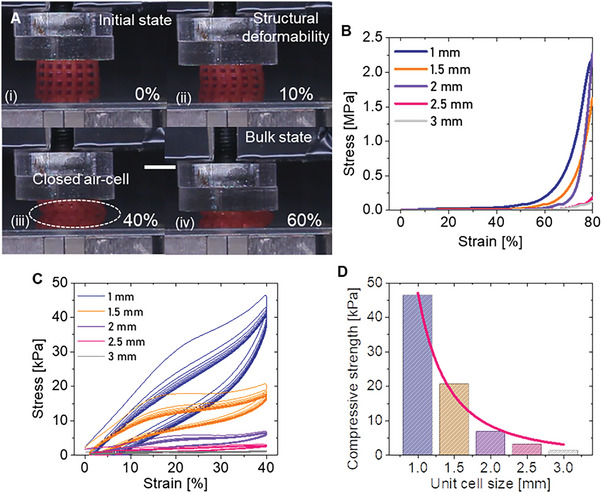
Compression test results. A) Sequential photographs of compression testing of a cylindrical sample with 2 × 2 × 2 mm unit cell, showing from left to right: (i) initial state; (ii) structural deformation (up to 40%); (iii) structural instability (white dot circle indicates closed air‐cells) at 40% compression; and (iv) bulk state at 60% compression (scale bar is 5 mm). B) Compression testing results for cylindrical samples having unit cells ranging from 1 × 1 × 1 to 3 × 3 × 3 mm, with steps of 0.5 mm, respectively. C) Cyclic compression testing results showing stress versus strain at 40% of compression. D) Compressive strength versus unit cell sizes.

In general, hyperelastic materials do not allow for volumetric changes, and thus they are nearly incompressible. However, due to the topologically transformed tessellations, with the proposed approach the 3D printed porous objects are capable of structural deformation (**Figure** **3**), including both positive and negative strains (Movie S1, Supporting Information). As shown in Figure 3A,B, all the objects can contract up to 80%. Interestingly, their stress responses increase monotonically with the applied strain. From a structural instability point of view, the stress response of architected materials is generally non‐monotonic and could fluctuate, or includes a negative stiffness regime after buckling^[^ ^20^ ^]^ (also called bi‐ or multi‐stability).

The emulsion ink‐based elastic lattice shows a unique compressive behavior, similar to elastomeric foam.^[^ ^21^ ^]^ The mechanical response can be described in three regimes (as observed in open‐cell foam^[^ ^22^ ^]^): a) linear elastic; b) plateau; and, c) densification. Structural deformation is dominant in the linear elastic and plateau regimes, and thus the elastic lattice can be compressed until the stress response reaches the densification regime. However, once entering this regime, the stress response increases exponentially. In this regime, regardless of unit grid size, all pores are closed (Figure 3A[iv]), and then an exponential behavior occurs above all due to the incompressibility of the constitutive material.

Accordingly, as shown in Figure S2A–E, Supporting Information, structural deformability with respect to the unit air cell size is evaluated at different compression ratios. For small unit cell sizes (1 and 2 mm) structural compressibility results up to 40% (see the cylindrical sample with 2 mm unit cells deformed at 10% and 40%, Figure S2C, Supporting Information). For larger sizes (2.5 and 3 mm), structural compressibility is up to 60%. Furthermore, at 40% compression, a wide range of compressive strengths, from 46.4 to 1.32 kPa can be achieved (Figure 3D). Similar to the previous findings for extensibility, the smaller the unit cell size, the larger the hysteresis (up to 28%).

From a reliability point of view, a compressive strain or stress does not provoke material failures. Indeed, the cyclic loading/unloading compression test demonstrated that reversible compressibility is equivalent to maximum compressibility. In addition, structural deformation of up to 40% shows the high potential for design life without material failures (i.e., 8000 cycles, see Supporting Information text and Figure S3, Supporting Information).

### 2.2 Monolithic Soft Actuators with Programmed Motions

Programmed motions are obtained by designing soft actuators consisting of two main parts: 1) a core made of elastic lattice with globally or locally distributed stiffness, and 2) an enveloping skin. The tessellated core is shaped like a bellow and has a specific distribution of macropores’ dimension that allows for the desired kinematic trajectories. The elastomeric skin plays a crucial role since it encases and seals the core structure while avoiding structural instability due to its geometry, therefore its convolution profile and thickness need to be suitably designed.

We investigated two promising skin convolution profiles via FEM simulation—V‐shape and U‐Shape—which can contract and elongate, as shown in Figure S4, Supporting Information. The V‐shape shows high extensibility up to 33% at 10 kPa. However, the U‐shape is extendible up to 26% at the same pressure, which corresponds to 12% less extensibility than the V‐shape. At a vacuum pressure of −2.5 kPa, both V‐ and U‐shapes are capable of −30% contraction. Based on these findings, the V‐shape convolution profile was utilized for fabricating the PELAs.

**Figure 4:**
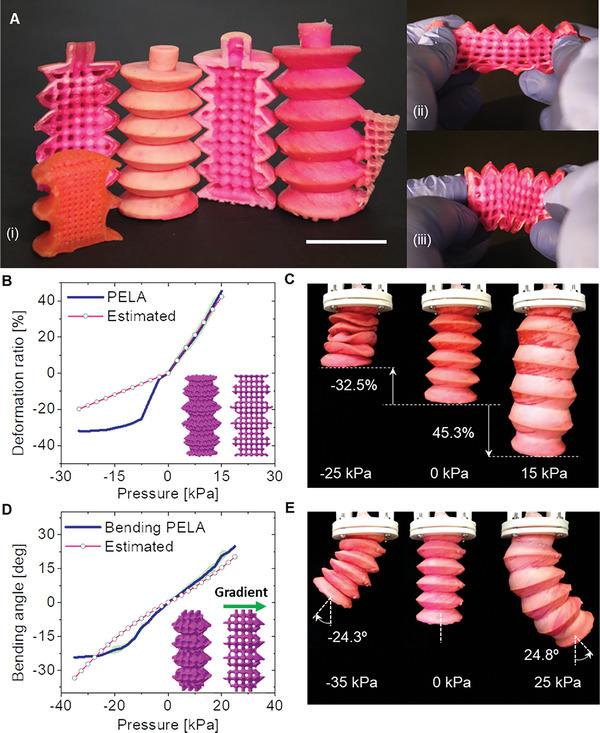
Pneumatic elastic lattice actuator (PELA). A) (i) (Left to right) cross sections and entire prototypes of biaxial longitudinal PELA, bending PELA, and bending PELA with a compliant layer; (ii) an asymmetric PELA being manually stretched, and (iii) compressed, showing different deformability at opposite sides. Scale bar is 30 mm. B) Quasi static characteristics of the biaxial PELA with 2 mm unit air cell size. C) PELA performing biaxial movements (−32% at −25 kPa and 45% at 15 kPa). D) Quasi static characteristics of the bidirectional bending PELA with stiffness gradient along transversal direction (unit air cells with dimensions from 3 × 3 × 3 mm to 1 × 1 × 1 mm). E) PELA performing bidirectional bending movements (−24.3 deg at −35 kPa and 24.8 deg at 25 kPa). In (B) and (D), experimental (blue line) and theoretical results (pink line) are shown.

One of the design and fabrication challenges of this approach consists in obtaining repeatable and reliable 3D printed structures with minimum skin thickness. We found that a skin thickness below 1 mm does not ensure safe operations under the pressure needed to induce movement (i.e., 25 kPa) without failure (see Figure S5, Supporting Information). On the other hand, 1.5 mm ensures a reliable fabrication (i.e., structure failure is avoided). Once the overall geometrical parameters of the skin have been identified, actuators with various core architectures and topologies are fabricated by a one‐step 3D printing (see **Figure** **4**A).

#### 2.2.1 Biaxial PELA

A purely axial movement is programmed by designing the PELA core with a symmetric stiffness distribution along the actuator (uniform grid tessellation). A quasi‐static characterization of the produced PELA was performed, together with an analytical model (Figure 4B,C and Movie S2, Supporting Information).

The symmetric grid structure has air cells with 2 × 2 × 2 mm and a pillar diameter of 1 mm, which result in a contraction ratio of 32% at −25 kPa, and an extension ratio of 45%, at 15 kPa, respectively. For the biaxial movement, 13% and 18% hysteresis are observed upon vacuum and positive pressure, respectively. As shown in Figure S6A, Supporting Information, the blocking force of the biaxial PELA goes from 4.87 N at 15 kPa to −3 N at −25 kPa. The strain response upon positive pressure shows a linear behavior and is in good agreement with the numerical model (Equation (1)), that is, 6.8% error at full elongation phase. This indicates that the axial stiffness of the PELA is strongly influenced by the presence of the core lattice structure, and that its equivalent stiffness exhibits a linear behavior when an external force is imposed. On the other hand, the strain response upon vacuum pressure shows a significant error (38% at full contraction phase). This is mainly due to the viscoelastic (nonlinear) behavior of the lattice. Indeed, once the core structure is subjected to the compressive strain, the air cells undergo the buckling, resulting in nonlinear (i.e., viscoelastic) behavior.

#### 2.2.2 Bending PELA

Bidirectional bending movement can be programmed by designing the PELA core with asymmetrical stiffness distribution along one transversal axis of the actuator (graded unit tessellation). As shown in Figure 4D,E, the result of quasi‐static characterization shows that by applying a negative or positive pressure, the asymmetric PELA either contracts and bends clockwise, or extends and bends counterclockwise, respectively (Movie S3, Supporting Information). Specifically, the bending angle measured at the distal end of the actuator is up to ≈25 deg at 25 kPa. For a vacuum pressure of −35 kPa, the bending angle reaches −24 deg. For the bidirectional bending movements, 16% and 21% hysteresis are observed upon vacuum and positive pressure, respectively. The blocking force of the bending PELA goes from 0.27 N at 25 kPa to −0.16 N at −35 kPa (see Figure S6B, Supporting Information). In principle, in this design configuration the strain rate varies gradually along the chosen transversal axis, causing bending. For example, when the core structure is under vacuum pressure, the unit cells become fully compressed, not allowing air passage, and similarly to a bulk structure, the actuator is incompressible.

In contrast with the biaxial PELA, the bending PELA shows a good agreement between the experimental and theoretical results (average 14% and 1.5% errors at vacuum and positive pressures). This is because 1) the bending angle is a nonlinear function of the imposed pressure (Equation (2)), and 2) the bending PELA is composed of different unit cells (3, 2, 1.5, and 1 mm). Regarding the latter, each unit cell has different critical loads inducing buckling. Consequently, the unit cells exhibit different strains along the vertical (row) direction of the core structure, while undergoing buckling at different compressive strains.

We believe that our design principle enabling bending movements is unique compared to conventional design principles (e.g., antagonistic bending and employing strain limiting layer). During movement, the bending PELA undergoes completely tensile or compressive strain, upon positive or negative pressure, respectively. Consequently, both the equivalent stiffness and deformability can be tuned through the correct sizing of the unit cells.

### 2.3 Gripper with Bending PELA with a Compliant Layer

Based on the promising results of the elastic lattice, and the programmability of its mechanical performance, we implemented a proof‐of‐concept of a new type of soft gripper. This gripper enables an adjustable opening angle via bidirectional movements and a compliant interaction with objects of various shapes.

The robotic platform with a programmed structure was built by integrating three modified PELAs, each designed to ensure a good trade‐off between deformability and conformability to objects for successful grasping. Thus, an external lattice structure is embedded to minimize any kinematic interference during bending while accommodating highly compliant interaction with objects that is obtained through the compressed lattice. The passive deformation of the external lattice structure is induced by the bending movement of the actuator. The gripping strength depends on the resistance to bending, and on the reaction force due to the external lattice structure. To release both effects, the bending PELA with a compliant layer bends in the opposite direction, and the opening distance of the gripper is thus adjustable.

**Figure 5:**
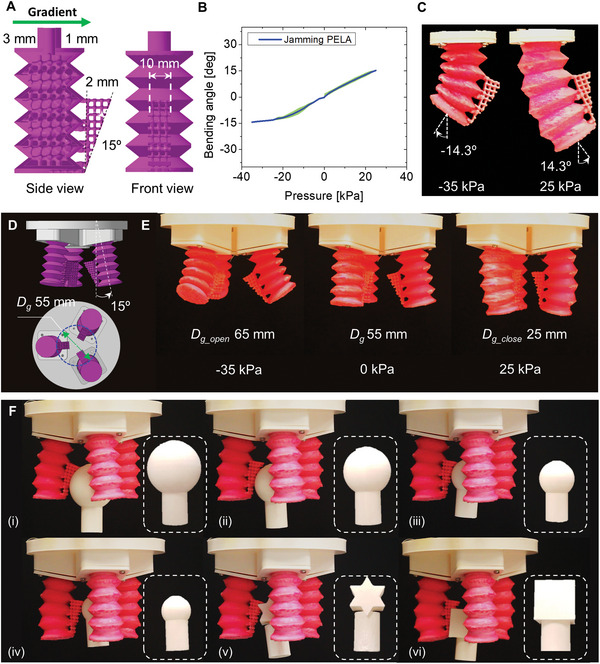
Design of three fingered gripper with external lattice structure. A) Design of the bending PELA with a compliant layer. B) Quasi‐static characteristics of, and C) the bending PELA with a compliant layer upon vacuum pressure (−35 kPa) and positive pressure (25 kPa). D) 3D CAD design of the three fingered gripper platform. F) Adjustable opening distance ranging from 65 mm (at vacuum pressure of −35 kPa) to 25 mm (at positive pressure of 25 kPa). G) High success rate in grasping with respect to objects with different configuration and dimensions; (i)–(iv) sphere with different radius from 40 to 24.5 mm, (v) star shaped object (width of 24.5 mm), and (vi) cubic‐shaped object (side length of 25 mm).

Given that mechanical performance is strongly influenced by the unit cell size, we found that the 2 mm unit cell is optimal, in terms of reliability, compressibility, extensibility, volumetric density, and stiffness (see Figure S7, Supporting Information). As shown in **Figure** **5**A, the valley space between convolutions is not filled with material, since doing so interferes significantly with the PELA bending motion.

The external lattice structure is designed with a trapezoidal shape so that it can adapt to objects of different shapes and sizes. As a result of quasi‐static characterization, the bending PELA with a compliant layer exhibits bidirectional movements as shown in Figure 5B,C (−14.3 deg at −35 kPa, 14.3 deg at 25 kPa). Interestingly, the bending PELA with a compliant layer can generate higher forces (−0.17 N at −35 kPa, 1.13 N at 25 kPa) than the bending PELA (see Figure S6B,C, Supporting Information). This is mainly due to the presence of the external lattice structure which improves the bending stiffness of the actuator when under positive pressure.

In Figure 5D, a three‐fingered gripper is presented. The starting opening distance is 55 mm by orienting each bending PELA with a compliant layer at 15 deg. During a grasping task, the bidirectional bending enables adjustable apertures with diameters ranging from 65 mm (at −35 kPa) to 25 mm (at 25 kPa), as shown in Figure 5E. In Movie S4, Supporting Information, the gripper shows high adaptability to spherical objects with diameters ranging from 24.5 to 40 mm (see Figure 5F[i–iv]). In addition, due to the presence of the compliant layer, both the gripper and the objects with different configurations (Figure 5F[v,vi]) can be interlocked with an enlarged contact surface. The three‐fingered gripper has 300 gf payload.

### 2.4 Programmed Multidimensional Continuum Actuator

**Figure 6:**
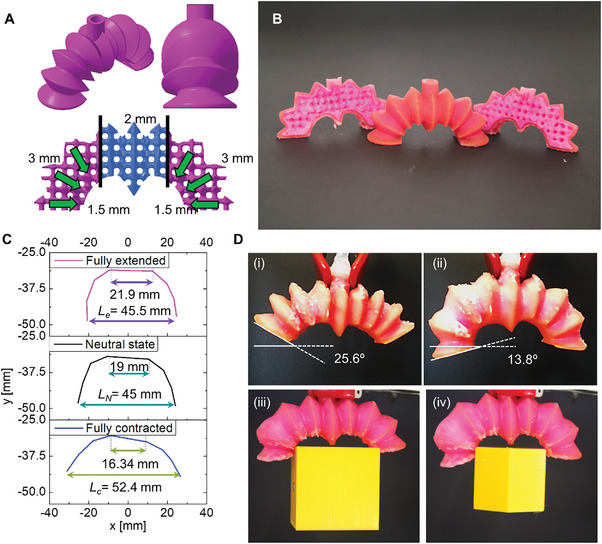
Continuum actuator encoding biaxial and bidirectional bending movements. A) (Top) Actuator skin. (Bottom) Core tessellated structure of the actuator, with gradient dimensions of unit air cells, which enables biaxial and bidirectional bending movements. B) Cross section of the fully printed actuator. C) Experimental results showing fully extended state under positive pressure, neutral state at atmospheric pressure, and fully compressed state upon vacuum pressure. D) The continuum structure (i) when fully contracted under vacuum pressure, and (ii) fully extended under positive pressure. Two grasping tasks achieved with (iii) cube of 40 mm at 15 kPa and (iv) hexagons of 35 mm at 17 kPa.

The programmed architecture shown in **Figure** **6**A,B enables synchronous bending and axial movements with only one fixed support, and a single actuation source.

Both distal ends of the actuator perform bidirectional movements, at the same the mid‐interface exhibits longitudinal deformation (i.e., contraction and elongation) at local level (Movie S5, Supporting Information).

As with the bending PELA introduced in Section 2.2.2, the bending movements are obtained by employing a gradient stiffness, that is, unit cells with side sizes ranging from 1.5 to 3 mm along the perpendicular direction of the convolution. In parallel, the longitudinal movements are obtained by a middle longitudinal structure similar to the biaxial PELA (in blue in Figure 6A), which mechanically connects the two distal asymmetric structures without forming any joints. The middle architecture has a symmetric geometry (2 mm unit cells) and uniform stiffness. Both bending and longitudinal structures are smoothly merged and interfaced by applying different blend distances.

We further investigated the trajectory at the distal ends of the actuator (Figure 6C). The bending angle can be obtained up to 13.8 deg upon positive pressure and up to 25.6 deg under negative pressure (see Figure 6D[i,ii]). As a result of instantaneous movements (i.e., bending and longitudinal deformations), the distance between the distal ends of the actuator varies, ranging from 52.4 to 45.5 mm. The longitudinal deformations in the middle exhibit 15% extension and 14% contraction upon positive and negative pressures, respectively (Movie S5, Supporting Information). Due to these kinematic movements (i.e., like a four‐bar linkage system), the multidimensional actuator can grasp wide objects (e.g., a cube of 40 mm and a hexagon of 35 mm), as shown in Figure 6D[iii,iv].

## 3 Discussion

The results of this work highlight that new generations of soft pneumatic actuators with no joints can be obtained by harnessing porosity in the materials (microporosity) and design (unit grid tessellation). For the materials, this is possible through 3D printing of emulsions which are precursors for stretchable foam structures. For the design, volumetric tessellation is key to producing continuum and complex architectures with smooth stiffness gradients.

The elasticity of the bulk material determines the deformability of pneumatic‐driven 3D architectures. The high compliance and strength of objects resulting from the emulsion ink material (i.e., high extensibility of up to 520% and tensile strength of 1.57 MPa) are mainly due to the micropores. These micropores mean that a much higher deformation can be obtained in the bending PELA compared to the same structure without microporosity in the unit cell walls (see Supporting Information text and Figure S8, Supporting Information).

Furthermore, the axial stiffness, and compressive strength can be tuned by tailoring the dimensions of the air cells. Through the cyclic tests, we found that a tensile strain of 150% and a compressive strain of 40% guarantee high design life and safety, while avoiding material failures. In the three‐fingered gripper (Figure 4) both the interlocking and highly compliant interaction with the object are employed.^[^ ^23^ ^]^ The structure of the actuators could be programmed to produce a bending motion useful to fully engage with the object, and integrate at their surface the external lattice structures that are passively compliant. We believe that this provides a proof‐of‐concept of a new soft gripper capable of a compliant interaction with objects of various shapes.

In comparison to literature focused on technologies enabling programmable deformations through metamaterials,^[^ ^4^, ^6^, ^7^, ^13^, ^24^ ^]^ as summarized in Table S3, Supporting Information, our design strategies based on elastic lattices offer unique characteristics, leading to the development of novel pneumatic actuators capable of a high deformation ratio with lightweight. Moreover, the elastic lattice implements movements at a relatively low‐pressure range, that is, the blocking force is up to 1.13 N at 25 kPa for the bending PELA with compliant layer. These aspects are important for achieving a high power‐to‐weight ratio (corresponding force‐to‐unit mass). Indeed, the PELAs’ weights are only 15 g, and thus high force to weight ratios (324.7 and 75.3 N kg^−1^ for biaxial PELA and bending PELA with compliant layer, respectively) are obtained in the monolithic structures. In contrast to multimaterial‐based approaches that are usually pursued by interfacing materials with different mechanical characteristics,^[^ ^4c^ ^]^ the presented design principles do not induce an increase in weight and material failures (i.e., delamination). Also, the presented design strategies are competitive compared to origami/kirigami technologies that have been widely used to achieve high force‐to‐weight ratio.^[^ ^24d^ ^–^ ^g^ ^]^ Although a design principle based on mechanical instabilities may be hindered by interferences that limit the actuator strokes, determining the actuator's deformation ratio and stiffness is attributed to the design of elastic lattice. In other words, unless the pillars and nodes of the unit cells are fully collapsed or fail, the elastic lattice contributes to achieving the desired movements of the actuator. Overall, while only few designs using metamaterials have addressed bidirectional deformability to date, it is worth noting that the presented elastic lattices enable a high deformation ratio of 77% at linear actuation and bidirectional bending that ranges from −14.3° to 14.3° (Figure 4C). We exploited these promising capabilities to develop the versatile soft gripper with adjustable opening distance and the multidimensional actuator. In particular, the kinematic synthesis using the elastic lattice opens up a new paradigm in designing continuum actuators capable of programmed deformations, for utilization in robotic applications.

The main potential of the new approach is that new continuum soft robots can be designed based on the 3D printing of graded stiffness structures. In articulated robotic systems, to increase the opening distance between the jaws of a gripper, joints and/or linkages are added producing increased degrees of freedom. Our soft continuum architecture encoded both axial and bending movements, specifically extension, contraction, and bidirectional bending, all in one printed structure and with just one pneumatic source.

The use of mechanical continuity will allow the robots to move and deform in a way that is more similar to living organisms (i.e., elephants). This makes them useful for tasks that require a high degree of adaptability and flexibility, such as exploration in unknown environments in, for example, search and rescue operations. Thus, the design principles introduced in this work can be exploited to explore new ways of integrating computing capabilities into soft machines by programming complex motions.

In this vision, we believe that our approach could be implemented to mimic natural continuum structures (e.g., elephant trunks) thus giving rise to totally new soft robots such as soft versatile manipulators. The muscular synergies of the artificial trunk could be implemented by encoding programmed modalities via intrinsic kinematic variables.^[^ ^3a^ ^]^

Our strategy could be exploited to scale the architecture by increasing the number of unit grid tessellations and their unit length. Therefore, achieving large‐scale robots (e.g., meter scale) will be possible with large‐scale 3D printers. Porosity‐based strategies could enhance the design of bioinspired mechanics by mimicking the anisotropy of the muscular arrangements. In addition, robotic design paradigms could move toward a robust soft continuum approach rather than articulated solutions.

## 4 Experimental Section

### Material Formulation

The 3D printing material formulation was composed of UV polymerizable monomers in a (W/O) emulsion type that could be converted into a solid upon light irradiation. The continuous phase of the emulsion (“oil”) was composed of the monomers: polyurethane acrylate (Ebecryl 8413, Allnex), epoxy aliphatic acrylate (Ebecryl 113, Allnex) at a 1:1 weight ratio, a photoinitiator which was a mixture of bis(2,4,6‐trimethylbenzoyl)‐phenylphosphineoxide (Igracure 184, BASF), and 1‐hydroxy‐cyclohexyl‐phenyl‐ketone (Igracure 819, BASF) at a 2:1 weight ratio, respectively. The total concentration of the photoinitiators was 2 wt% of the emulsion continuous phase. The continuous phase was prepared by mechanically mixing (RW20 digital, IKA) at 10 000 RPM the monomers and photoinitiators at 60 °C, until a homogeneous formulation was achieved.

The dispersed phase of the emulsion was composed of triple distilled water, while Pluronic L‐121 (Sigma Aldrich) was used as the emulsifier, and hydroquinone (99%, Sigma Aldrich), sulforhodamine B sodium salt (BioReagent, Sigma Aldrich), and ABIL EM 90 (Evonik) were used as additives.

To prepare the printing composition, Pluronic L‐121 and hydroquinone were dissolved in the continuous phase at a concentration of 4 wt% and 0.05 wt%, respectively. Separately, sulforhodamine B was dissolved in TWD at 0.005 wt%. To mix the phases, the dispersed phase was dropwise added while mixing at 6000 RPM. Then, for the coarse emulsion, the solution was homogenized (S25N‐18G rotor, T25 Ultra Turrax homogenizer, IKA) at 13 000 RPM for 10 min. Last, Abil EM 90 was added to the emulsion at 1 wt%. To remove air before the 3D printing, the emulsion was mixed at a defoaming mode in a THINKY mixer for 2 min.

A Freeform Pico Plus39 DLP printer (ASIGA, Australia) was used for the 3D printing. The printing was performed at 0.2 mm layer thickness with 1.2 s UV irradiation at *λ* = 385 nm. After printing, the objects were rinsed with acetone and water, followed by post curing under UV light (365 nm) for 15 min. Finally, to create the micropores (diameter: 1–10 µm), the water was evaporated by heating the object at 110 °C for 1 h. The samples without micropores (results in Figure 2B and Figure S7, Supporting Information) were fabricated by the same process but without the last step.

### Different Design Strategies for 3D Architected Materials

The 3D architected material was obtained by the volumetric tessellations from the bulk structure. Herein, the volumetric tessellation was generally composed of regular or irregular unit cells (see Figure S9, Supporting Information). While the former represented a hierarchical unit cell composed of descriptive and homogeneous geometric patterns such as equilateral triangles or squares, the latter indicated non‐homogeneous geometric patterns. From a mechanical point of view, the regularity and irregularity in unit cells were important design parameters, determining whether the 3D architected materials exhibited isotropic or anisotropic deformations. More importantly, regular tessellations could also induce the anisotropic deformations by tuning either, 1) Poisson's ratio based on their geometry (e.g., interfaced angles where nodes and pillars were engaged), or 2) varying stiffness (e.g., modifying diameter [or thickness] of pillars).

The fabrication by printing 3D architected materials was challenging mainly due to the supporting structures and material failures during printing. According to the complexity of the geometric patterns, the supporting structure could be avoided. Given that the DLP printing created a 2D layer simultaneously across the entire layer, the regular unit tessellation was beneficial in terms of addressing both issues arising from the 3D printing and ensuring high printability. In this work, considering homogeneity and printability, the grid tessellation was selected (see Figure S10, Supporting Information). A gradient stiffness was achieved by transforming the topology of the node and the diameter of pillar which enabled different unit cells to be smoothly merged.

### Analytical Modeling for Pneumatically Driven 3D Architected Materials

In principle, the entangled air cells represented compliant mechanics consisting of a finite number of coupled joints (nodes) and flexure hinges (pillars). Thus, an equivalent stiffness determined the kinematic movements of the overall structure, which could be predicted as a 3D spring mass system (also called spring‐mass lattice). In the approach presented in this work, deformations at each node and pillar occurred mainly due to the pressure imposed. To analyze the kinematics of pneumatically driven 3D architected materials, explicit mathematical approaches were introduced.

Symmetrically distributed air cells induced an isotropic deformation. Given that pneumatic inflation or deflation incurred omnidirectional deformations of the structure, it should be noted that for the symmetric grid structure, deformations in *x* and *y* (or *z*) axes were equivalent (i.e., isotropic, ∆*x* = ∆*y* = ∆*z*), while the asymmetric grid structure underwent anisotropic (or orthotropic) deformations (i.e., ∆*x* ≠ ∆*y* ≠ ∆*z*). Here, deformations on the *xy* plane were considered and desired movements (mainly biaxial and bidirectional movements) were identified by analyzing the equivalent stiffness.

To better understand this, first it was assumed that the air cells had a linear relation with the resulting strains. Then, either, isotropic or anisotropic deformations were enabled according to the distributed equivalent stiffness, and a schematic diagram of each node and pillar is depicted in Figure S10, Supporting Information.

### Symmetric versus Asymmetric Grid Structures

The symmetric grid structure accommodating axial movements consisted of uniformly distributed unit cells (Figure S11C,D, Supporting Information), which could be conceived as an equivalent spring in parallel ($k_{\mathrm{eq},\;i}=\sum_{i=1}^{n}k_{i,j}$). This was because the axial strains occurring in each row and column were equivalent for entire unit cells. Herein, the equivalent stiffness for elongation (*k*_ex*i*,*j*_) and contraction (*k*_cont*i*,*j*_) could be varied according to the unit cell size.

The force acting on the cross section (*A*_e_ on the *xz* plane) was equal to the force induced by the imposed pressure (*F* = *PA*_e_). To allow for biaxial deformation, a bellow skin was employed, thus the cross section was determined by the mean diameter of the actuator (*D*_m_ = (*D* *+* *D*_in_)/2). The cross section was given by ($A_{e}=\frac{\pi }{4}D_{m}^{2}$). Therefore, an analytical expression for the steady state elongation and contraction could be expressed by:

(1) $$\in_{\mathrm{longitudinal}}\left(P\right)=\frac{PA_{e}}{L_{i}\cdot \mathrm{sign}\left(P\right)}\;\mathrm{With}\;\mathrm{sign}\;\left(P\right)=\left\{\begin{array}{c} \sum_{i=1}^{n}k_{@{\mathrm{ex}}_{i,j}},\;P\geq 0\\ \sum_{i=1}^{n}k_{@{\mathrm{cont}}_{i,j}},\;P<0 \end{array}\right.$$

On the other hand, given that asymmetric grid structures induced an anisotropic deformation, the bending movements could be obtained by distributing the stiffness along the lateral direction of the actuator (Figure S11E,F, Supporting Information). To understand this better, the Euler Bernoulli principle was employed, and the radius of curvature of the actuator (1/*R*) could be approximated by $\frac{1}{R}\approx \frac{M}{EI}$, where *R*, *E*, and *I* were the radius of curvature, modulus of elasticity, and area (second) moment inertia of the deflected actuator, respectively. Assuming that the curvature was constant, the bending angle of the actuator was then a function of the length (*θ* = *L*/*R*). When the actuator bent in a steady state, the new length (*L*) corresponded to the sum of the initial length (*L*_i_) and deformed length (*δL*_ss_) (i.e.*, L* = *L*_i_ + *δL*_ss_). Given that the deflection of the actuator (*δL*_ss_) corresponded to the deflection owing to the total equivalent stiffness and imposed pressure, then the bending angle of the actuator was the second order of imposed pressure, that is, $\theta \left(P\right)=\frac{L}{R}=\frac{PA_{e}\cdot e}{EI}\cdot \left(L_{i}+\frac{PA_{e}}{k_{\mathrm{eq}}}\right)$, where *e* was an eccentric distance offset from the neutral axis of the actuator.

The equivalent stiffness in bending movements was subjected to both, parallel, and series connections. First, as shown in Figure S11E,F, Supporting Information, the different unit cells were distributed in a lateral direction (i.e.*, j*th column), while the unit cells along each row direction were uniform (i.e.*, i*th row). A total equivalent stiffness was analyzed, by assuming that the equivalent stiffness along the columns was decoupled (*δL*_*i*1_ ≠ *δL*_*i*2_… ≠ *δL_ij_*) whereas it was coupled along the rows (*δL*_1*j*_ = *δL*_2*j*_… = *δL_ij_*), respectively.

Thus, the total equivalent stiffness (i.e., bending stiffness) could be simplified, as $k_{\mathrm{eq}}=\frac{1}{\sum_{j=1}^{m}\frac{1}{{\left(k_{\mathrm{eq},i}\right)}_{j}}}.$ Similar to Equation (1), considering an equivalent stiffness for both, compressive, and tensile strains, an analytical expression for the steady state bending could be expressed by:

(2) $$\begin{array}{ccc} \theta \left(P\right) & = & \frac{L}{R}=\frac{PA_{e}\cdot e}{EI}\cdot \left(L_{i}+\frac{PA_{e}}{\mathrm{sign}\left(P\right)}\right)\;\mathrm{With}\;\mathrm{sign}\\ \left(P\right) & = & \left\{\begin{array}{c} \frac{1}{\sum_{j=1}^{m}\frac{1}{{\left(k_{\mathrm{eq}@\mathrm{elong},i}\right)}_{j}}},\;P\geq 0\\ \frac{1}{\sum_{j=1}^{m}\frac{1}{{\left(k_{\mathrm{eq}@\mathrm{cont},i}\right)}_{j}}},\;P<0 \end{array}\right. \end{array}$$

This follows that the bending angle was a nonlinear function of the imposed pressure, and it should be noted that this model was built based on assumptions—1) linear elasticity of the unit cells upon compressive and tensile strains, 2) the cross section of the actuator was perpendicular to the neutral axis during deformation bending, and iii) deformations in the lattice that could possibly occur in the transverse direction were neglected, focusing on longitudinal strains.

### Bellow Skin Convolution Profiles

Among the possible designs, the bellow type was a unique structure made of multiple convolutions periodically arranged along the vertical direction of the structure. Its mechanical performance was determined by geometrical parameters (i.e., number of convolutions, means diameter, and total length) and material characteristics (i.e., Young's modulus). More importantly, convoluted profiles of the bellow had shown a high potential, that is, producing a large contraction ratio, preventing buckling in vacuum‐based foam actuation,^[^ ^6c^ ^]^ and biaxial movements in hyperelastic actuators.^[^ ^25^ ^]^ In this approach, two design parameters were investigated—the skin thickness and the shape of the convolution profile.

The former was related to the airtightness required in the final actuator resulting from the 3D printing process.^[^ ^26^ ^]^ In the proposed design, the axial stiffness of the core lattice architecture should be dominant, with respect to the skin. In fact, programming the lattice architecture was the key design phase for obtaining the asymmetric stiffness distributions needed to achieve different kinds of movement. In principle, the axial stiffness of the skin was proportional to *t^3^*, where *t* was the skin thickness.^[^ ^27^ ^]^ Therefore, by employing a minimum skin thickness, a significant decrease in axial stiffness could be obtained. Considering the printability and airtightness of the skin layer, different skin thicknesses were investigated and interfaced with the core lattice structure.

Meanwhile, the latter was related to determining the deformability while avoiding the mechanical instability. Bellow skins with different convolution profiles (V‐ and U‐shapes) were investigated by a FEM.

The V‐ and U‐shapes of the convolution profiles were designed as shown in Figure S4, Supporting Information (where *h*_u_ and *h*_v_ represent the height of the single convolution and were 12.6 and 10 mm, respectively, *r* = 6 mm, and *θ* = 77.3°). The outer diameter (*D*) was fixed at 29 mm, and the inner diameters (*D*_in,v_ and *D*_in,u_) of each type of convolution were 18 and 15.5 mm, respectively. These design parameters followed a preliminary study that was carried out (UH‐PAM made of U‐shape convolution)^[^ ^6c^ ^]^ and the hyperelastic bellow which Digumiri et al., introduced.^[^ ^25^ ^]^ The material properties of the skin were defined by the incompressible neo‐Hookean hyperelastic model (listed in Table S1, Supporting Information). A FE model was created in ANSYS 19.2 (ANSYS Inc., Canonsburg, PA, USA) for static structural analysis, in order to evaluate the deformation versus imposed pressure.

### Tensile and Compression Specimens

Tensile specimens along ASTM‐D‐638 were designed with unit air cell sizes ranging from 1 × 1 × 1 mm to 3 × 3 × 3 mm with steps of 0.5 mm (See Figure S12, Supporting Information). Herein, two design parameters (i.e., the diameter of the pillar and number of air cells per unit volume) were used. Cylindrical samples were designed for compression tests with a diameter of 16 mm and length of 9.4 mm. By applying a unit grid tessellation, different unit air cells ranging from 1 × 1 × 1 to 3 × 3 × 3 mm with steps of 0.5 × 0.5 × 0.5 mm were obtained. For all samples, the pillar diameter was fixed to 1 mm, and then the number of air cells per unit volume was varied to obtain different unit sizes. In addition to this, a blended distance (also called fillet) was fixed to 0.5 mm to merge the node (i.e., flexure rib) smoothly. The tensile and compression tests were performed using a universal material testing machine (Z005, Zwick/Roell, Ulm, Germany). For the tensile and compression tests, a preload of 0.1 N and a velocity of 200 mm min^−1^ were applied.

### Design of Pneumatic Actuators and Control Setups

The pneumatic actuators and grippers were designed by 3D modeling based on SolidWorks CAD software (Dassault Systems, US) considering the 3D printing building volume of 31 mm × 49 mm × 72 mm. An assembly product function was used to obtain the skin and internal structure by topological transformation. Both files were imported in Autodesk Netfabb for the additive manufacturing program (Autodesk, Netfabb 2021, US), which transformed and topologically optimized the 3D objects. For the internal structure, the unit tessellation was defined by the unit topology function by the lattice command provided by Netfabb. Different tessellations were implemented with the unit air cell side ranging from 1 to 3 mm. To obtain the programmed architecture, a gradient thickness was applied so that each row or column in the grid tessellation had different unit cells. Finally, the internal lattice structure was interfaced with the skin, and then the final stereolithography files were exported.

To characterize the pneumatic actuators, supporting structures were fabricated using a 3D printer (Ultimaker S3, Ultimaker, Netherlands) with tough white ABS (acrylonitrile butadiene styrene. The vision tracking system built on an open‐source physics Java framework (Tracker, Open‐source program), and the AURORA electromagnetic tracking system (AURORA EM) with 6‐DoFs electromagnetic localization probe (AURORA EM, Northern Digital Inc., Waterloo, Canada), were both exploited to investigate kinematic trajectories. The pressure control was created using flow regulators embedded in a pressure feedback system: a vacuum flow regulator (ITV0090‐3BS, SMC corporation, Japan) providing vacuum pressure control ranging from 0 to −100 kPa, and an electro‐pneumatic flow regulator (ITV0010‐3BL, SMC corporation, Japan) providing positive pressure control ranging from 0 to 100 kPa. A LABVIEW program was developed for data generation and acquisition of an analog signal for the pneumatic pressure control, and for the acquisition of the pressure sensing output through DAQ6218 (National Instruments, TX, US).

## Conflict of Interest

The authors declare no conflict of interest.

## Author Contributions

S.J. and O.B. contributed equally to this work. L.B. and S.J. conceived the concept. L.B. and S.M. supervised the project. S.J. carried out simulations. O.B. carried out experiments on the material and printing. O.B. fabricated the protoypes. S.M. guided the material and printing process. S.J. carried out experiments and analyzed the data. L.B. and S.J. wrote the manuscript. L.B., S.J., O.B., and S.M. reviewed and edited the manuscript.

## Supporting information

Supporting Information

Supplemental Movie 1

Supplemental Movie 2

Supplemental Movie 3

Supplemental Movie 4

Supplemental Movie 5

## Data Availability

The data that support the findings of this study are available in the supplementary material of this article.
