# Supplementary material for: Jointless Bioinspired Soft Robotics by Harnessing Micro and Macroporosity
Source: Adv Sci (Weinh). 2023 Jun 15;10(23):2302080. doi: 10.1002/advs.202302080 (PMC10427402; doi:10.1002/advs.202302080)
Supplement: Supplementary file 1 — Supporting Information [file ADVS-10-2302080-s003.pdf]

ADVANCED SCIENCE
Open Access

Supporting Information

for *Adv. Sci.*, DOI 10.1002/advs.202302080

Jointless Bioinspired Soft Robotics by Harnessing Micro and Macroporosity

Seonggun Joe, Ouriel Bliah, Shlomo Magdassi and Lucia Beccai**

Supporting Information

Jointless Bioinspired Soft Robotics by Harnessing Micro and Macroporosity

Seonggun Joe, Ouriel Bliah, Shlomo Magdassi, and Lucia Beccai

Constitutive modeling of μEI

The stress-strain curves show a hyperelastic behavior, similar to rubber like elastomeric materials. In general, such elastic constitutive models can be represented by their fundamental relation of the strain-energy potential versus principal strains (or stretches). To understand better on continuum mechanics, the elastic strain energy should be defined, as follow:

$$\delta U = \int \sigma : \delta\varepsilon \, dV \qquad \text{(s1)}$$

where σ and ε are the Cauchy stress and strain, and the strain energy density can be written by

$$\partial\Psi = \sigma : \delta\varepsilon \qquad \text{(s2)}$$

also, it can be represented by

$$\sigma = \frac{\partial\Psi}{\partial\varepsilon} \qquad \text{(s3)}$$

Given the deformation gradient $(\mathbf{F} = \partial x/\partial X)$, the Cauchy-Green deformation tensor (C) is then $\mathbf{F^T} \cdot \mathbf{F}$, thereby the invariants of C can be expressed:

$$I_1 = tr(\boldsymbol{C}) = \lambda_1^2 + \lambda_2^2 + \lambda_3^2$$

$$I_2 = \left(tr(\boldsymbol{C})^2 - tr(\boldsymbol{C}^2)\right) = \lambda_1^2\lambda_2^2 + \lambda_2^2\lambda_3^2 + V_1^2\lambda_3^2 \qquad \text{(s4)}$$

$$I_3 = \det(\boldsymbol{C}) = \lambda_1^2\lambda_2^2\lambda_3^2$$

where the principal stretch (λ) is represented by a ratio of deformed length (l_d) to the initial length (l_0). For hyperelastic materials, different strain energy density functions are available

(e.g., Mooney-Rivlin, Neo-Hookean, Ogden models, etc.). They are commonly built from two key features – Isotropic and Isochoric (incompressible) materials. Hence, the deformation gradient (F) and invariants of C are

$$\mathrm{F} = \begin{bmatrix} \lambda & 0 & 0 \\ 0 & \frac{1}{\sqrt{\lambda}} & 0 \\ 0 & 0 & \frac{1}{\sqrt{\lambda}} \end{bmatrix} \tag{s5}$$

$$I_1 = \frac{2}{\lambda} + \lambda^2$$

$$I_2 = 2\lambda + \frac{1}{\lambda^2} \tag{s6}$$

$$I_3 = 1$$

For uniaxial tensile test, the stress can be defined, as a function on the stretch [1]:

$$\sigma = 2\left(\lambda^2 - \frac{1}{\lambda}\right)\left(\frac{\partial\Psi}{\partial I_1} + \frac{1}{\lambda}\frac{\partial\Psi}{\partial I_2}\right) \tag{s7}$$

To investigate the best parameters ensuring good agreement for experimental results, Mooney-Rivlin (M-R) and Neo-Hookean (N-H) models were employed. For both models, the strain energy density functions are defined, as follows:

$$\Psi_{\mathrm{MR}} = \sum_{i,j=0}^{N} C_{ij}(I_1 - 3)^i (I_2 - 3)^j + \sum_{k=1}^{M} D_k (J - 1)^{2k} \tag{s8}$$

$$\Psi_{\mathrm{NH}} = C_1(I_1 - 3) \tag{s9}$$

The material constants obtained by different constitutive models are summarized in Table. S1.

Table S1. Material constants obtained by constitutive hyperelastic modeling

Model	Parameter	Mean [Pa]	Residual	Incompressibility parameter (D1), [Pa^-1]
Mooney Rivlin – 2 par	C10	99125.39	15.158	0
	C01	-1404.28		
Mooney Rivlin – 3 par	C10	19431.474	2.357	0
	C01	116092.187		
	C11	5631.378		
Mooney Rivlin – 5 par	C10	70657.411	1.1412	0
	C01	51672.975		
	C11	-5330.239		
	C20	1801.245		
	C02	2051.469		
Neo-Hookean	C10	197265.280	15.165	0

Extensibility test results

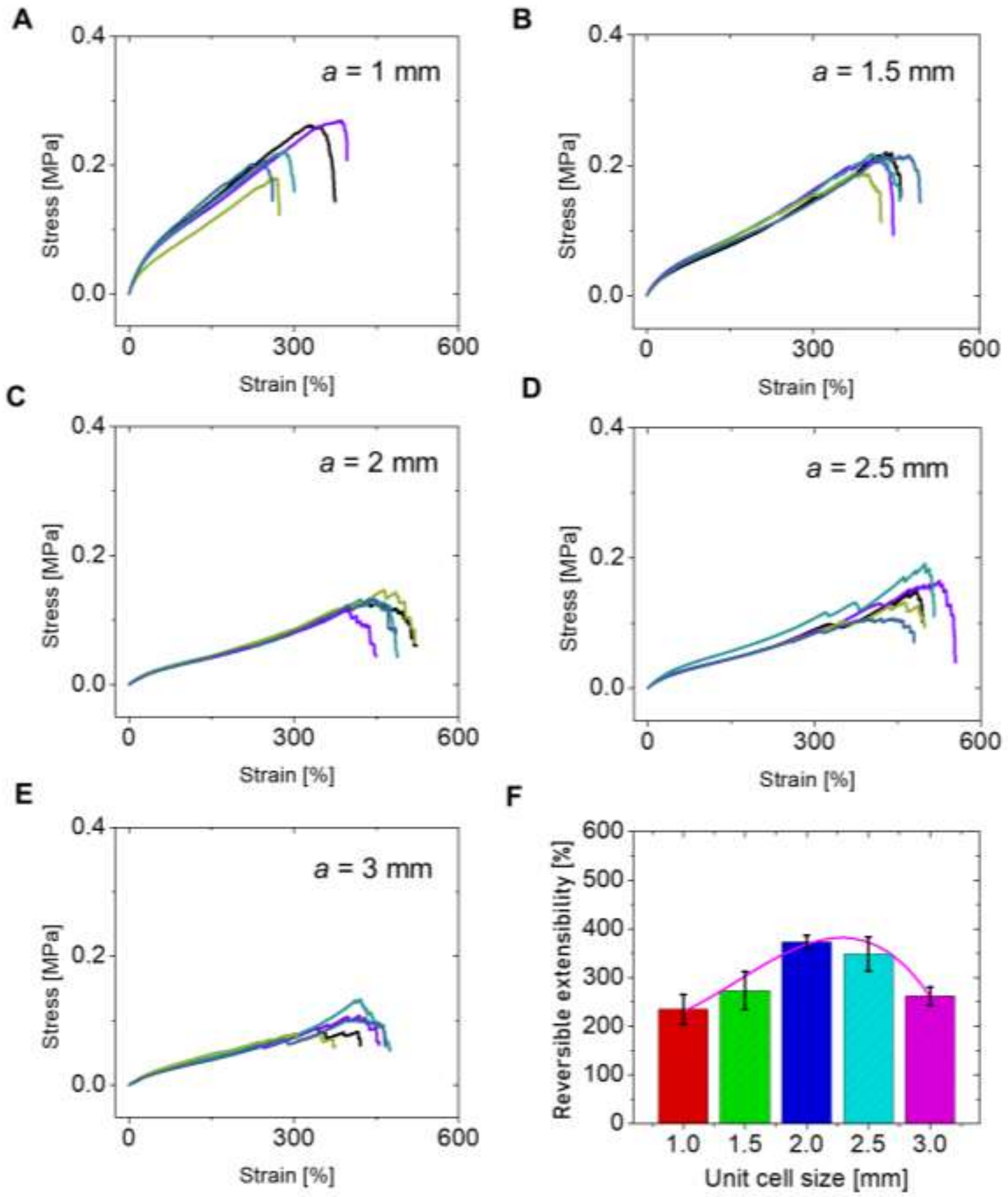

Figure S1. Stress responses of different unit cells ranging from (A) 1 mm to (E) 3mm. (F) Reversible extensibility versus unit cell size.

Table S2. Mechanical characteristics obtained by tensile tests

	Bulk with micropores	Unit cell dimension				
		1 mm	1.5 mm	2 mm	2.5 mm	3 mm
Max. extensibility [%]	523.09 (STD 27.17)	321.32 (STD 61.11)	454.2 (STD 25.55)	483.50 (STD 24.54)	508.480 (STD 28.49)	438.28 (STD 41.05)
Reversible extensibility [%]	-	234.64 (STD 30.455)	273.39 (STD 38.99)	373.53 (STD 13.29)	348.55 (STD 35.0)	261.83 (STD 18.34)
100% Modulus [MPa]	0.32 (STD 0.013)	0.11 (STD 0.011)	0.064 (STD 0.003)	0.036 (STD 0.0029)	0.036 (STD 0.004)	0.032 (STD 0.002)
Tensile stress [MPa]	1.57 (STD 0.066)	0.23 (STD 0.038)	0.210 (STD 0.013)	0.15 (STD 0.013)	0.15 (STD 0.003)	0.103 (STD 0.02)
Number of breaks	1	8.2 (STD 0.89)	15.6 (STD 2.049)	6.2 (STD 1.77)	8.6 (STD 1.97)	9.8 (STD 1.57)
Yield stress [MPa]	1.54 (STD 0.057)	0.19 (STD 0.024)	0.140 (STD 0.0160)	0.12 (STD 0.01)	0.11 (STD 0.013)	0.066 (STD 0.0039)

Compressibility test results

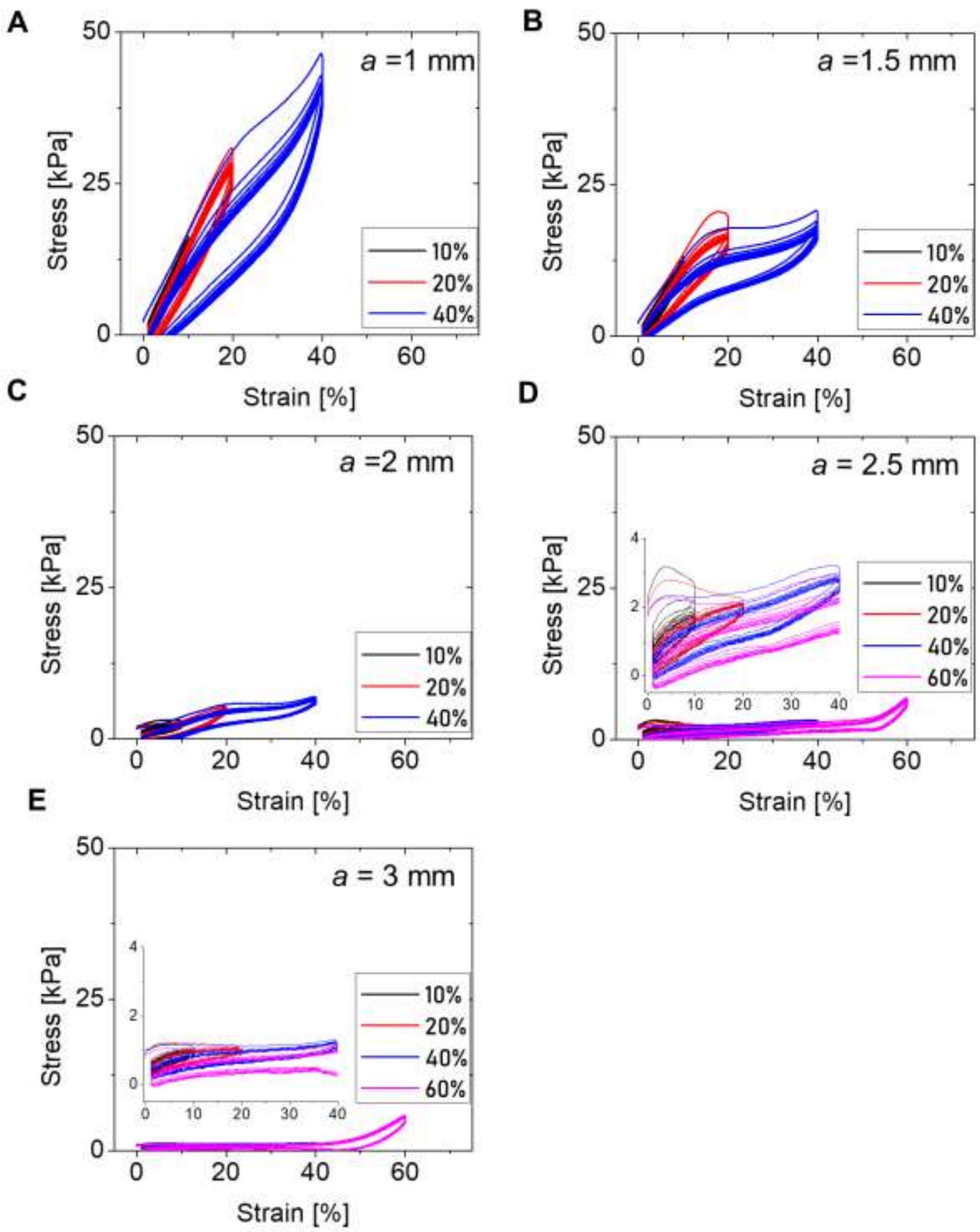

Figure S2. Cyclic compression test results with respect to different unit cell sizes, ranging from (A) 1mm to (E) 3 mm.

Cyclic test analysis

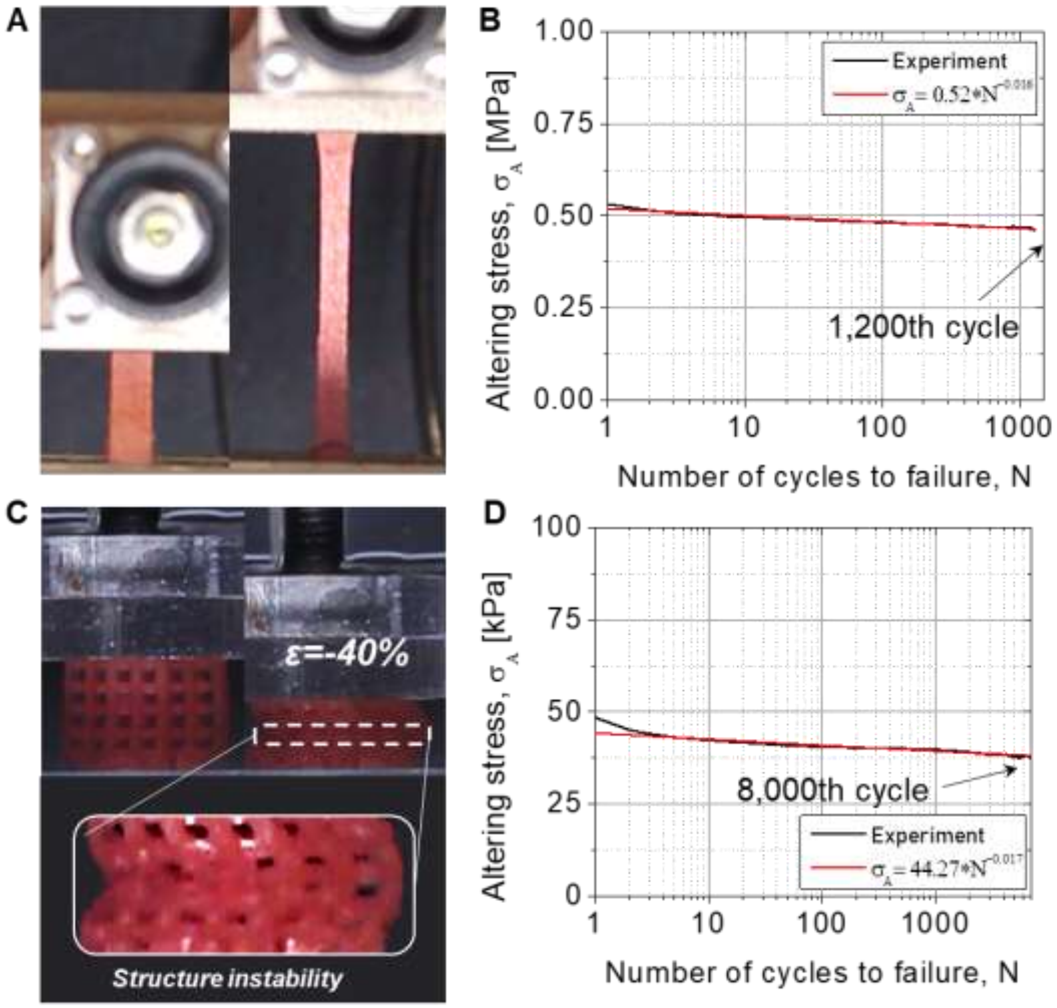

Figure S3. Stress versus number of cycles to failure. (A) Photographs of cyclic tensile test. (B) Cyclic tensile test up to 1,200 cycles with 150% strain. (C) Photographs of cyclic compression test and structure instability with 40% compressive strain. (D) Cyclic compression tests up to 8,000 cycles with 40% strain.

Despite promising findings at high reversible extensibility of the μEI, the elastic lattice structure could be delicate due to material failures. Notably, given that the pneumatic actuation induces an omnidirectional deformation, the flexure rib of each air cell undergoes high stress concentration, which may result in a fracture during inflation. Therefore, understanding and predicting the behavior of elastomeric system during their lifetime are crucial in order to realize reliable robotic applications (e.g., PELA).

To the best our knowledge, analyzing and/or predicting such soft materials' damages relies on experimental studies, similarly reported in [2]. In contrast to conventional reliability analysis (i.e., rigid materials), the mechanical fracture in soft materials can be addressed from both microscopic and macroscopic viewpoints. From the microscopic point of view, rubber like materials generally undergo the stress softening (also called Mullins effect) when they subjected to loading/unloading cycles [3]. Once they are unloaded to the stress-free state, the unloading curve is lower than the loading curve with a relevant hysteresis. Such phenomenon appears in the first (few) cycles, then it generally disappears after a few more cycles (2 – 10 cycles) if the imposed strain keeps constant [4]. This is because the shorter chains in the rubber matrix are torn from the filter, resulting in no longer resisting deformations. From the macroscopic point of view, pioneering studies identified that the damages due to repetitive deformations could be significantly accumulated, resulting in residual strain or stress. Moreover, it is worth to mention that the energy dissipation during cyclic loading could cause permanent (irreversible) damage. For these reasons, mechanical performances (i.e., stress response) could undergo a stiffness reduction or increase of stress softening [5].

In our approach, tensile and compressive strains that could induce a damage are experimentally investigated. The cyclic loading/unloading tests were performed by exploiting a universal material testing machine (Z005, Zwick/Roell, Ulm, Germany). For the cyclic tensile test, 1,200 cycles were performed for 12 hours. A tensile strain of 150 % was imposed. With cylindrical sample made of 2x2 macropores, the cyclic compression test was performed by running 8,000 cycles for 24 hours. A compressive strain of 40% was imposed. For both experiments, the pre-load-setting was 1 N, and the velocity of 200 mm/min was employed.

As a result, both cyclic tensile and compressive tests show the stress softening after the first cycle. Comparing the first with the second cycle, the tensile stress decreases up to 3% (from 0.532 MPa to 0.516 MPa), and the compressive stress reduces up to 7% (from 48.49 kPa to 45.11 kPa). We observed that both cyclic deformations lead to accumulative deformations. Thereby, the µEI undergoes gradual stress softening. Similar to traditional fatigue analysis, both curves were predicted by using the S-N curve formula ($\sigma = aN^{\alpha}$, a and α are constants) as a function of the number of cycles and fatigue stress. Both interpolated curves show a good agreement with the experimental values.

With these results at cyclic tensile and compressive tests, we defined a safe operation range for inflatable operation *via* positive pressure and proven that vacuum-driven actuation is completely safe. In detail, the tensile strain and stress need to be limited to 150% and 0.4 MPa in order to achieve a high design life. For the compressive strain and stress, there are no limits, yet note that the structure deformation (mainly due to compression) can be achieved within 40%.

Bellow skin convolution profile

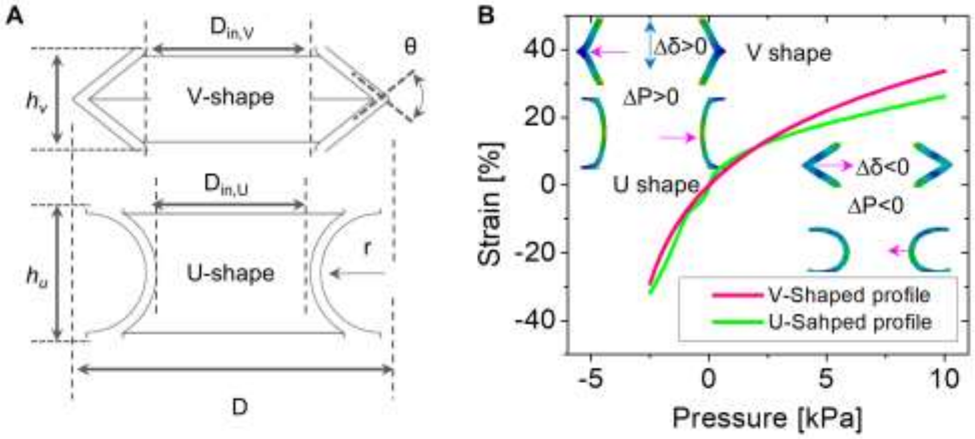

Figure S4. V- and U-shapes of convolution profiles. (A) Design parameters of U- and V-shapes. Diameter is fixed to 30 mm. (B) Finite Element Method (FEM) analysis.

Digital light processing printing

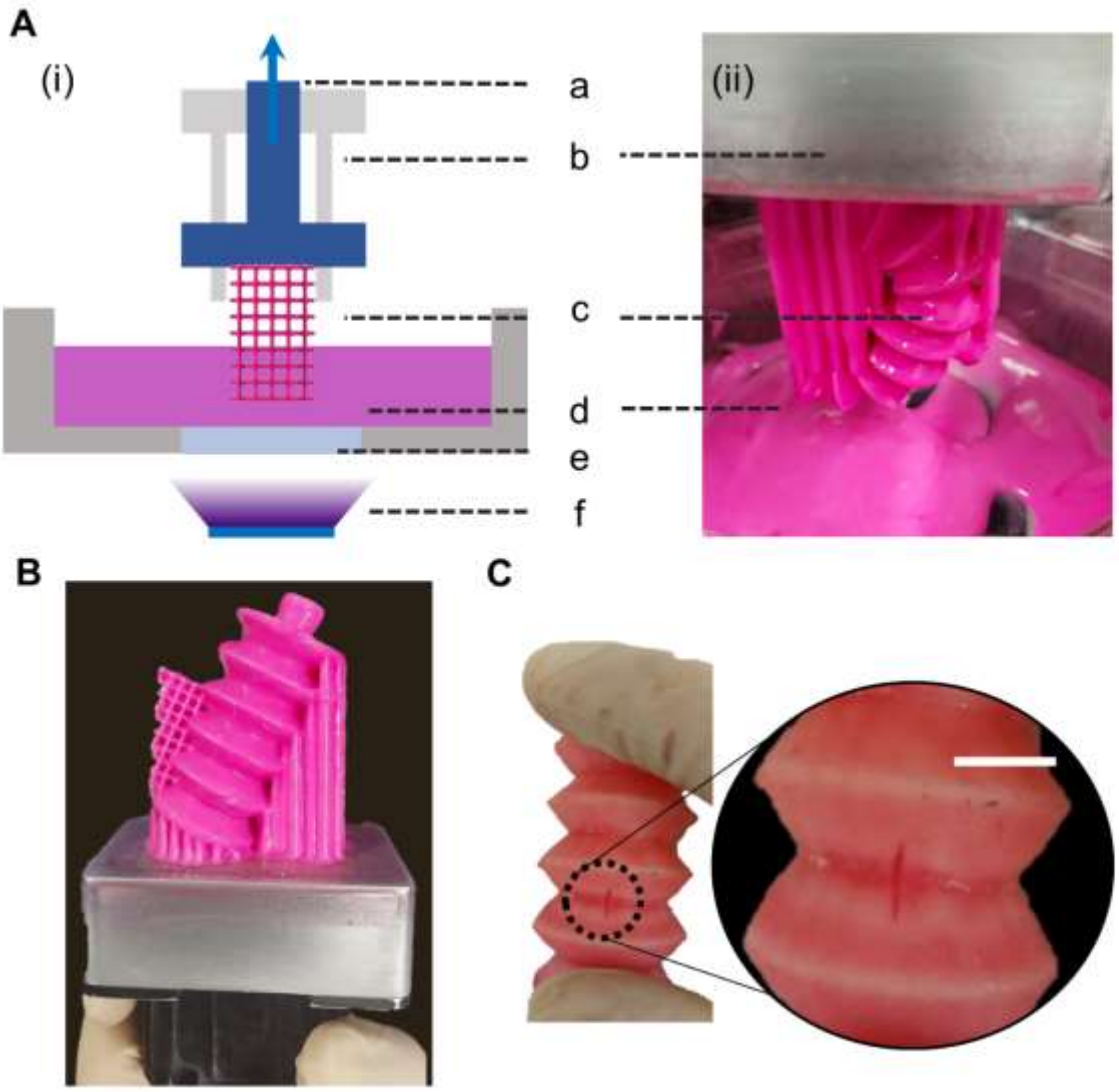

Figure S5. Digital Light Processing (DLP) printing technology. (A) Working principle of DLP: (i) schematic diagram and (ii) photograph. a: platform moving vertically, b: building plate, c: cured model, d: liquid resin, e: transparent vat bottom, f: UV lighting. (B) Printed model with supporter. (C) Material failure in the fabricated skin with 1 mm thickness due to airtightness imperfection (Scale bar: 10 mm).

Quasi-static characteristics of blocking force versus imposed pressure

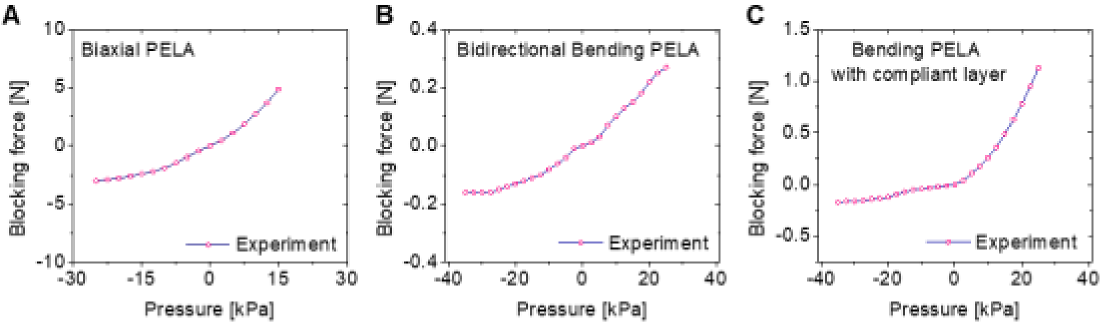

Figure S6. Graphs relative to the blocking force versus the applied pressure. (A) Biaxial PELA, (B) Bending PELA, and (C) Bending PELA with a compliant layer.

Qualitative analysis of different unit cells

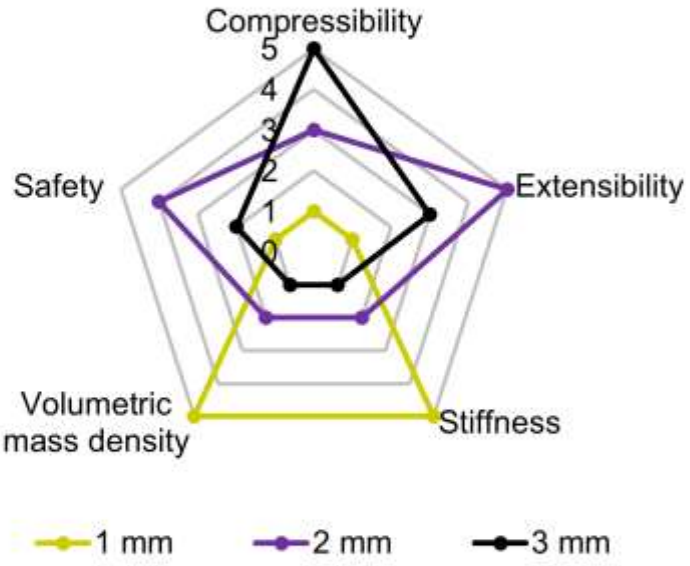

Figure S7. Radar chart with the legends indicating 0 (low) to 5 (high).

Comparative analysis of bending actuators

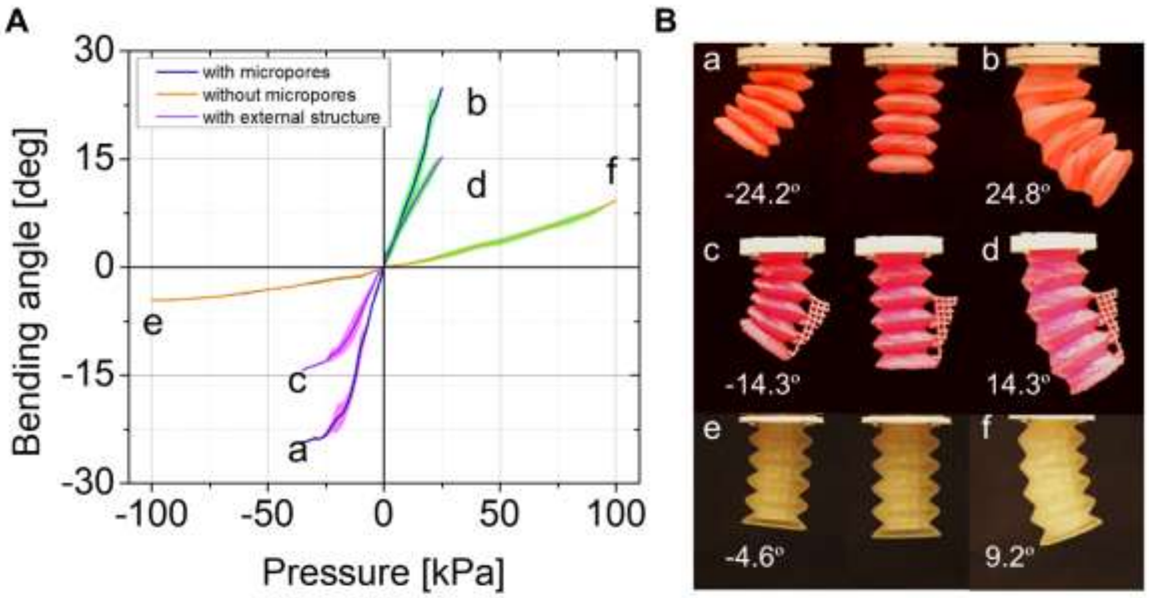

Figure S8. Comparative analysis for different actuators. (A) Graph relative to the bending angle vs. the applied pressure to obtain directional movements by different actuators (with vs. without micropores vs. with external lattice structure). (B) Different actuators at pressurized or depressurized state; (a) to (b) show variations of the angular trajectory achieved by the bending PELA; (b) to (d) show variations of the angular trajectories achieved by the bending PELA with a compliant layer; (e) to (f) show variations of the angular trajectories achieved by the bending actuator made from the polyurethane acrylic (without micropores).

Figure S7 shows bending angle responses of different bending actuators made of the μEI and Polyurethane acrylate (without micropores). The bending PELA (made of μEI) shows high compliance with a large bending angle ranging from -24.2 deg at -35 kPa to 24.8 deg at 25 kPa. In contrast, the bending actuator made of the polyurethane acrylate is relatively stiff. Indeed, to achieve the bending angle of 5 deg (CCW), high pressure of 70 kPa should be imposed, which corresponds to 10 times of the bending PELA (7.5 kPa). For the vacuum pressure, the bending actuator made of the polyurethane acrylate (without micropores) shows only -4.6 deg at -100 kPa. Given that the bending PELA could achieve the same angle at -2.5 kPa, the elasticity and deformability of the soft material play a vital role in determining the mechanical performance of lattice-based pneumatic actuators. Indeed, as the elasticity decreases, the compressibility of the architected material is significantly reduced, and thus the

structure is hardly compressible when subjected to vacuum pressure. Therefore, to achieve kinematic trajectories in enlarged scale, a high input power would be necessary.

Moreover, the bending PELA with a compliant layer (fig. S7c and d) shows a slight degradation in bending movements compared to the bending PELA. Indeed, at the positive pressure of 25 kPa, the bending angle of 14.3 deg (CCW) is achievable. Similarly, the bending angle of -14.3 deg (CW) is achieved at vacuum pressure of -35 kPa.

Different volumetric tessellations

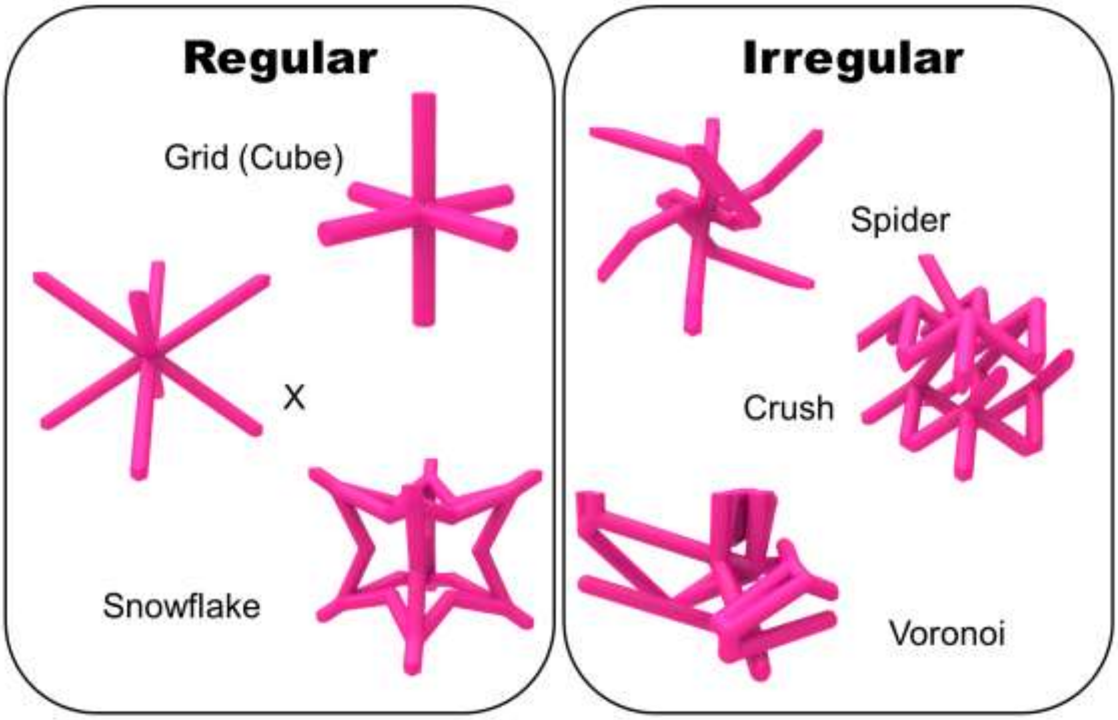

Figure S9. Different strategies for unit tessellation. Regular (grid, x, and snowflake) vs. Irregular (spider, crush, Voronoi).

Unit grid tessellations

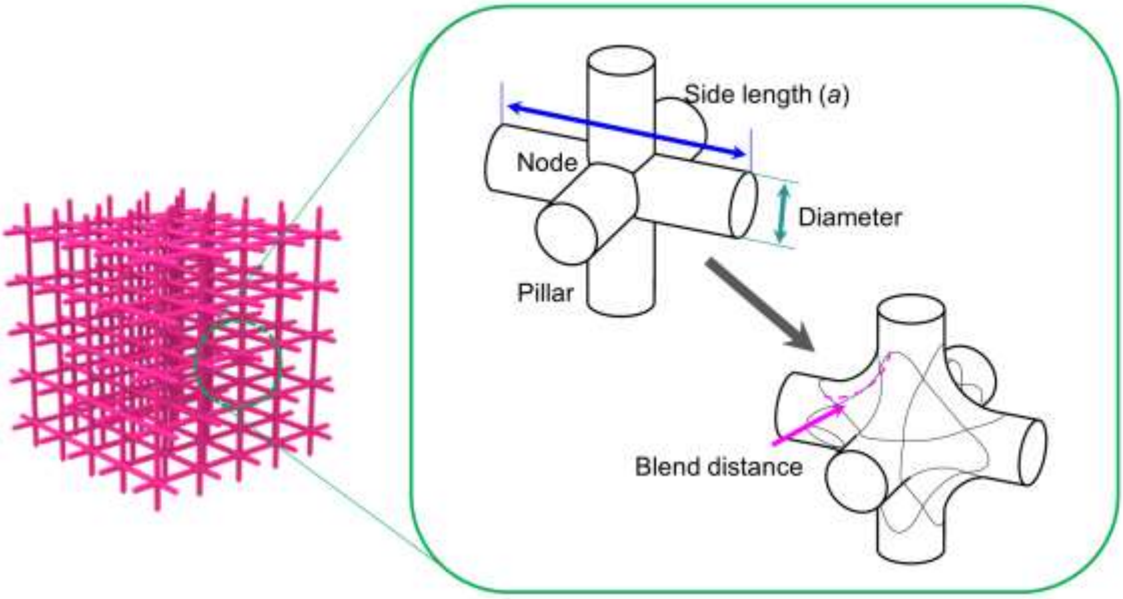

Figure S10. A topological transformation of the node by applying a blend distance.

Analytical model for grid tessellations

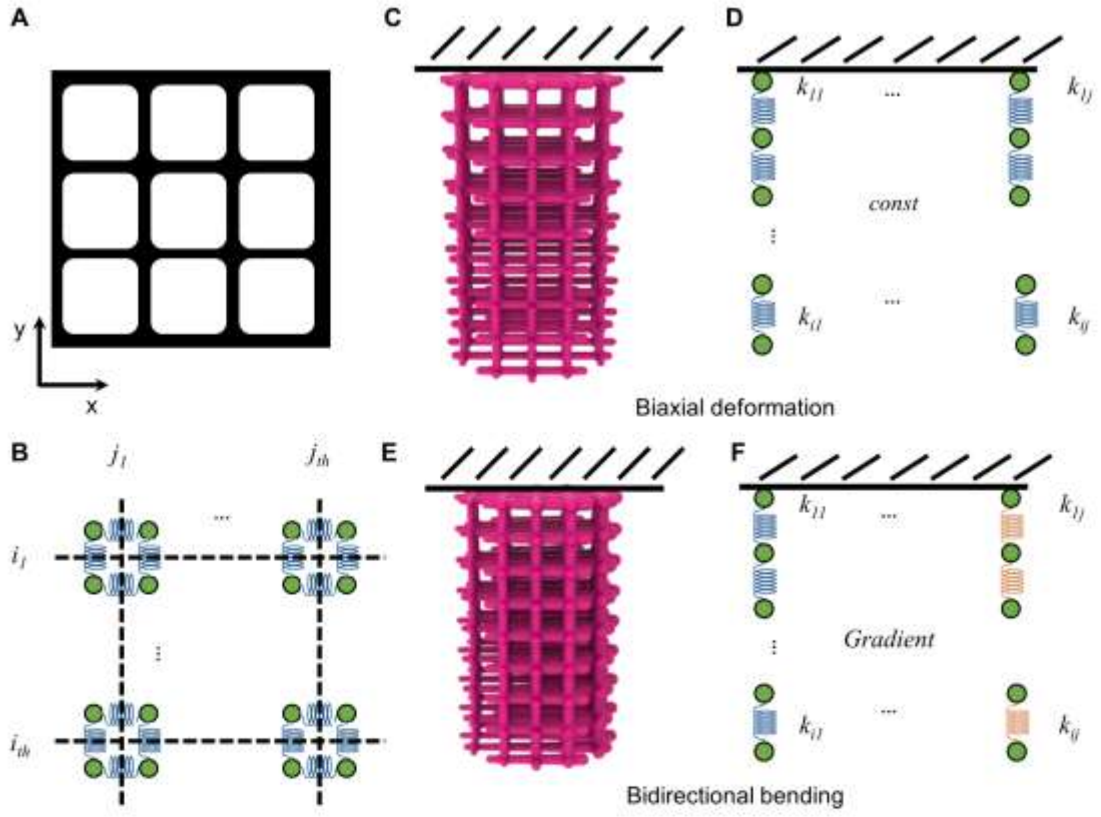

Figure S11. Analytical modelling for grid tessellation. (A) A 2D layer of the grid structure (i.e., 3x3 matrix). (B) The spring mass system for the grid structure. (C) Symmetric grid structure and (D) diagram composed of constant spring stiffness. (E) Asymmetric grid structure and (F) diagram composed of inconstant spring stiffness along the lateral direction.

Design of grid tessellated tensile and compression specimens

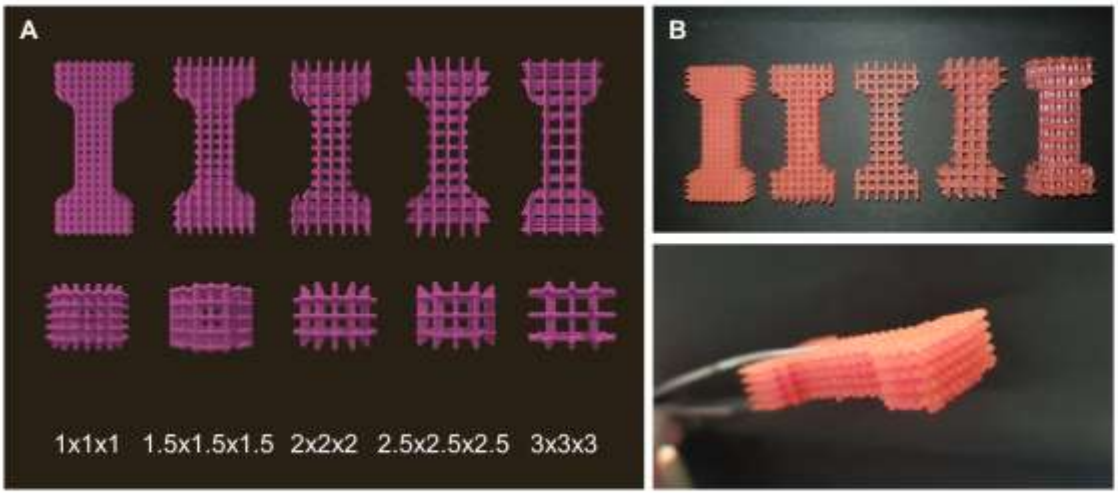

Figure S12. A design and fabrication of tensile and compression specimens. (A) 3D rendered design of tensile and compression specimens with different unit cells (ranging from 1 mm to 3 mm). (B) Fabricated tensile specimens with different air cells (top), and an isotropic view of tensile specimen with 1 mm (bottom).

Table S3. Comparative analysis of different design principles enabling programmable deformations via metamaterials

	Ref	Demonstrator	Deformation type	Deformation	Pressure imposed [kPa]	Blocking force [N]	Actuator weight [g]	Force to weight ratio [N/kg]
Mechanical instability	[6]	Linear	Uniaxial	-40%	-90	-	-	-
	[7]	Linear	Uniaxial	- 4 mm	-70	25	11.1	-
	[8]	Multidimensional	Unidirectional curvature	50 m^{-1}	23	-	100	-
	[9]	Linear	Biaxial	-	-	-2 to 15	-	-
Origami	[10]	Linear	Uniaxial	-50.8mm	-85	431.5	320	134
	[11]	Linear	Uniaxial	360 %	17	9.8	8.3	118
		Bending	Unidirectional	360º	25	-	-	-
		Multidimensional	-	-	-	-	-	-
	[12]	Linear	Uniaxial	-90%	-80	29.4	2.6	1153.8
		Bending	Unidirectional	-	-	-	-	-
		Multidimensional		-	-	-	-	-
Kirigami	[13]	Multidimensional		-	20	-	-	-
Multi-material based	[14]	Bending	Unidirectional	-	150	2.4	-	-
		Twisting	-	-	-	-	-	-
	[15]	Linear	Uniaxial	-8.5%	6	4.9	-	50
		Bending	Unidirectional	90º	6	-	-	-
		Twisting	-	225º	6	-	-	-
	[16]	Linear	Uniaxial	-	100	-	-	-
		Bending	Unidirectional	-	54	-	-	-
		Multidimensional		-	-	-	-	-

	[17]	Bending	Unidirectional	-	80	4	-	-
	[18]	Linear	Uniaxial	-	-	-	-	-
		Bending	Unidirectional	-	75	0.66	6.6	100
		Twisting	-	-	-	-	-	-
Elastic lattice	**Our work**	Linear	Biaxial	32%	-25	-3	15	324.7
				45%	15	4.87		
		Bidirectional bending	Bidirectional	-14.3º	-35	-0.17	15	75.3
				14.3º	25	1.13		
		Multidimensional		-14% contraction with 25.6º bending	-15	-	18	-
				15% contraction with 13.8º bending	17			

Movie Legends:

Movie S1. Compression and tensile tests for the unit air cell with 2x2x2 mm.

Movie S2. Biaxial Pneumatic Elastic Lattice Actuator (PELA) movements.

Movie S3. Bidirectional PELA movements.

Movie S4. Three fingered gripper composed of Bending PELAs with a compliant layer.

Movie S5. Multidimensional motions in continuum structure.

References

[1] M. S. Xavier, A. J. Fleming, Y. K. Yong, *Advanced Intelligent Systems* **2021**, *3* (2), 2000187.
[2] V. Morovati, A. Bahrololoumi, R. Dargazany, *International Journal of Plasticity* **2021**, *142*, 102993.
[3] a) L. Mullins, *Rubber chemistry and technology* **1969**, *42* (1), 339; b) J. Harwood, L. Mullins, A. R. Payne, *Journal of Applied Polymer Science* **1965**, *9* (9), 3011.
[4] B. Fazekas, T. J. Goda, *International Journal of Mechanical Sciences* **2021**, *210*, 106735.
[5] a) T. Zhang, S. Lin, H. Yuk, X. Zhao, *Extreme Mechanics Letters* **2015**, *4*, 1; b) W. Zhang, X. Liu, J. Wang, J. Tang, J. Hu, T. Lu, Z. Suo, *Engineering Fracture Mechanics* **2018**, *187*, 74.
[6] D. Yang, M. S. Verma, J. H. So, B. Mosadegh, C. Keplinger, B. Lee, F. Khashai, E. Lossner, Z. Suo, G. M. Whitesides, *Advanced Materials Technologies* **2016**, *1* (3), 1600055.
[7] M. A. Robertson, J. Paik, *Science Robotics* **2017**, *2* (9), eaan6357.
[8] R. V. Martinez, J. L. Branch, C. R. Fish, L. Jin, R. F. Shepherd, R. M. Nunes, Z. Suo, G. M. Whitesides, *Advanced materials* **2013**, *25* (2), 205.
[9] J. T. Overvelde, T. Kloek, J. J. D'haen, K. Bertoldi, *Proceedings of the National Academy of Sciences* **2015**, *112* (35), 10863.
[10] A. Zaghloul, G. M. Bone, in *Actuators* MDPI, **2023**, 72.
[11] R. V. Martinez, C. R. Fish, X. Chen, G. M. Whitesides, *Advanced functional materials* **2012**, *22* (7), 1376.
[12] S. Li, D. M. Vogt, D. Rus, R. J. Wood, *Proceedings of the National academy of Sciences* **2017**, *114* (50), 13132.
[13] L. Jin, A. E. Forte, B. Deng, A. Rafsanjani, K. Bertoldi, *Advanced Materials* **2020**, *32* (33), 2001863.
[14] L. Ding, N. Dai, X. Mu, S. Xie, X. Fan, D. Li, X. Cheng, *Materials & design* **2019**, *182*, 108000.
[15] M. Schaffner, J. A. Faber, L. Pianegonda, P. A. Rühs, F. Coulter, A. R. Studart, *Nature communications* **2018**, *9* (1), 1.

[16] F. Connolly, C. J. Walsh, K. Bertoldi, *Proceedings of the National Academy of Sciences* **2017**, *114* (1), 51.
[17] B. C. Mac Murray, X. An, S. S. Robinson, I. M. van Meerbeek, K. W. O'Brien, H. Zhao, R. F. Shepherd, *Advanced Materials* **2015**, *27* (41), 6334.
[18] N. Willemstein, H. van der Kooij, A. Sadeghi, *Soft matter* **2022**, *18* (38), 7269.
